# Dynamic Speed Adaptation for Path Tracking Based on Curvature Information and Speed Limits †

**DOI:** 10.3390/s17061383

**Published:** 2017-06-14

**Authors:** Citlalli Gámez Serna, Yassine Ruichek

**Affiliations:** Le2i FRE2005, CNRS, Arts et Métiers, University Bourgogne Franche-Comté, UTBM, F-90010 Belfort, France; yassine.ruichek@utbm.fr

**Keywords:** speed adaptation, speed limits, curve speed, path tracking, lateral error, road safety, autonomous vehicle

## Abstract

A critical concern of autonomous vehicles is safety. Different approaches have tried to enhance driving safety to reduce the number of fatal crashes and severe injuries. As an example, Intelligent Speed Adaptation (ISA) systems warn the driver when the vehicle exceeds the recommended speed limit. However, these systems only take into account fixed speed limits without considering factors like road geometry. In this paper, we consider road curvature with speed limits to automatically adjust vehicle’s speed with the ideal one through our proposed Dynamic Speed Adaptation (DSA) method. Furthermore, ‘curve analysis extraction’ and ‘speed limits database creation’ are also part of our contribution. An algorithm that analyzes GPS information off-line identifies high curvature segments and estimates the speed for each curve. The speed limit database contains information about the different speed limit zones for each traveled path. Our DSA senses speed limits and curves of the road using GPS information and ensures smooth speed transitions between current and ideal speeds. Through experimental simulations with different control algorithms on real and simulated datasets, we prove that our method is able to significantly reduce lateral errors on sharp curves, to respect speed limits and consequently increase safety and comfort for the passenger.

## 1. Introduction

Autonomous vehicle navigation has been a challenging field of study where today the main goal is to provide safety. In order to accomplish that, vehicle control has to perform accurate path tracking; in other words, it should minimize the lateral distance between the vehicle’s position and the defined path.

In real driving scenarios, speeding is one of the main causes for traffic accidents [1], which has been considered for Advance Driver Assistance Systems (ADAS). The correlation between speeding and lateral errors is positive, and as a consequence, safety-related issues will depend on vehicle’s speed control.

Intelligent Speed Adaptation (ISA) systems have been proven in several studies [2,3,4,5] to reduce accidents by respecting speed limits. However, there are several other factors that need to be considered for precise speed control. One of the major ones involves road geometry.

Analyzing road structure will permit the identification of straight and curved segments ahead while driving, allowing the lateral control system to adjust the vehicle’s speed accordingly. Curve detection is of vital importance since the crash rate is at least 1.5-times higher than in tangent (straight) segments [6]. In addition, when a curve becomes sharper, the number of accidents increases [7,8]. Hence, different studies have focused on analyzing how processing road structure [9,10,11] may benefit control systems.

Using speed limit information, systems like ISA will inform the driver when exceeding speed limits. On the other hand, approaches considering curvature information [9,10] will emit warnings when approaching the curve too fast. In any case, control systems require precise knowledge of the vehicle’s global position. This location information is usually provided by Global Positioning Systems (GPS), but its main drawback is that it suffers from big positioning errors. These errors are caused by different factors like blockage, multipath, etc., specially in urban environments. In order to overcome these big errors, different solutions proposed the use of additional sensors such stereo vision, IMU, radar or LiDAR [11,12,13,14]. For example, vision-based solutions together with digital maps are capable of estimating the ego motion of the vehicle and correct the vehicle’s global position using a map-matching algorithm [13]. In [14], the use of cameras, an accurate digital map, GPS and inertial measurements improve the ego-localization of the vehicle using a variant of the Kalman filter [15]. The main limitation of vision approaches is visibility, since shadows, highlights, occlusions or weather conditions affect the accuracy of data analysis. Moreover, IMU sensors are able to estimate the current position of the vehicle. Fusing GPS and IMU with the typically Kalman filter [15] provides absolute position and orientation, even if GPS data are not available all of the time [16].

Road map databases (GIS maps) together with GPS data provide accurate road geometry and localization [6,17]. An approach to analyze road geometry identifying critical curves where accidents occur frequently was proposed in [6]. Wang et al. [17] calculated the road ahead of the vehicle through a flexible road model. Nevertheless, GIS information is not always available, and GPS is starting to be accessible or will be soon for the general public. For this reason, the choice of GPS data for tracking approaches is convenient. Our work is based on Real-Time Kinematics GPS (RTK-GPS), which has the highest absolute position accuracy (up to a few centimeters), to implement an automatic approach that goes beyond warnings for adjusting the vehicle speed depending on the upcoming road characteristics, e.g., curves or speed limit zones.

**Figure 1:**
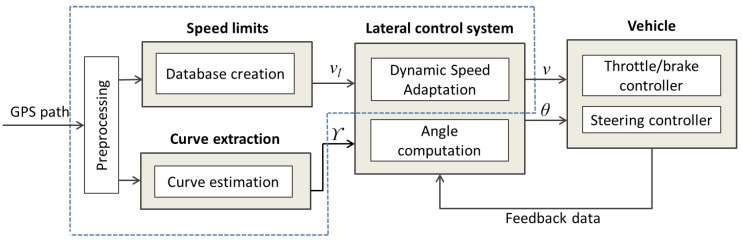
Overall workflow of the vehicle path tracking system. The blue dotted bounding box highlights our contributions.

This paper proposes a Dynamic Speed Adaptation (DSA) method for control systems, which together with speed limits database creation and curve extraction, adjusts automatically the vehicle’s speed (see Figure 1). These two approaches, speed limits database creation and curve extraction, are performed off-line including the preprocessing step. Their output provides the main parameters to be analyzed by the speed negotiation algorithm of DSA in order to compute the ideal speed. The identification of sharp curves is performed using GPS positions to define segments that belong to a curve or a tangent line. This is in order to estimate the convenient driving speed for the detected high curvature segments. The speed limit database is created for the traveled paths identifying the positions where speed limits change. As the tracking performance is evaluated through lateral errors, our DSA is tested in simulations with different steering control algorithms. On the modeling level, our proposed work would:

- preprocess the GPS path,
- extract position information of sharp curves and speed limit zones,
- compute the recommended speed for each sharp curve,
- obtain the speed limits for the path,
- analyze the current vehicle position in the traveled GPS path,
- compute the triggered distance at which the vehicle needs to start decelerating to ensure smooth speed transitions,
- adjust speed during triggered distance to respect sharp curved speed or speed limits,
- control the vehicle’s speed automatically.

The rest of this paper is organized as follows. Section 2 presents a review of related works. Section 3 provides an overview of the steering control algorithms that we used to test our approach. Section 4 explains our DSA method. In Section 5, we discuss and analyze the results obtained with and without considering automatic speed adaptation in the different steering control algorithms. The last section presents conclusions and highlights the main contributions to continue with future work directions.

## 2. Literature Overview

As our DSA method estimates the speed based on either curvature information or speed limits, works related to these two problems are considered.

### 2.1. Intelligent Speed Adaptation Systems

Intelligent Speed Adaptation (ISA) systems have been proven to reduce the number of injuries and fatal accidents as shown in studies conducted in the UK [2,5], Sweden [18], Belgium [4], Denmark [19,20], Australia [21], France [3], as well as in a study made by the European Union with the project Road Safety Data, Collection, Transfer and Analysis (DaCoTA) [1].

Utilizing ISA systems not only helps to reduce tragic crashes or injuries, but also: (1) gives the control system more time to identify and respond to hazards (unexpected events) [22]; and (2) reduces the driver’s need to monitor the speedometer with external speed limit signs. For young drivers, it increases their safety since their inexperience of sharing their attention while driving and respecting the information of the ISA system could lead to accidents [21].

It is also known that in order to take advantage of fully-controlled ISA systems, the speed limit information and the positioning system must be accurate [20]. For this reason, speed limit databases up to date with possible variations (time of the day, weather conditions, type of vehicle) are necessary. An approach to develop and use a speed limit database in the ISA warning system was implemented as a mobile application software for Australian roads [23].

Even though Google Maps’ application provides speed limits, it does not contain information regarding temporal situations (i.e., weather conditions, timing, construction areas) since it is an exhaustive work to maintain an updated database worldwide. Our speed limit database is based on static speed limits defined by the traffic speed regulations in France [24].

### 2.2. Curve Speed Estimation

The analysis of traffic accidents on curves has been a major concern of vehicle safety for several years [8]. No matter the efforts made to highlight dangerous curves with traffic signs [25], drivers are still surprised with the speed they need to reach to have an appropriate control over the steering wheel.

Nowadays, in the era of autonomous navigation, various solutions have been proposed to try to overcome this problem. One of them focused on analyzing human behavior while driving in curves. Lehtonen et al. [26] examined how drivers anticipate their eyes look-ahead fixations on curves to provide visual guidance for steering. Zhang et al. [27] proposed a driver speed model for curved roads based on the recorded behavior of the driver.

Other works based their curve speed computation on the road shape estimation, either for implementing it in semi-autonomous or autonomous solutions. Related to road geometry analysis, identification of curved or straight segments has been performed either by analyzing vehicle’s ahead motion [28,29,30] or by measuring physically-related parameters like curve radius, length and angle [6,7] using GPS and GIS information. Once these curves are identified, their corresponding speeds are calculated considering road friction and super-elevation angles [9,11,31].

Curve speed warning systems correspond to semi-autonomous solutions, which consist of informing the driver, through sound or display, about the recommended speed [9,18,32,33], e.g., Varhelyi [18] proposed a system that goes beyond curves, considering also factors such as wet roads and visibility conditions (darkness) to compute the appropriate speed and inform the driver visually or audibly. Alternatively, the warning signal may come through some force applied to the throttle (accelerator) to prevent speeding. That is the case of Huth et al. [34], who proposed a curve warning system for motorcyclists through a force feedback throttle or a haptic glove.

Adaptive Cruise Control (ACC) systems, which are another type of semi-autonomous solution, have also considered curve speeds together with speed limits to adjust velocity respecting headway distance to the lead vehicle [10].

Concerning autonomous solutions, it is not a surprise that the automotive industry is trying to develop control systems that consider every possible situation to drive safely. Nevertheless, user acceptance is still a factor to overcome in order to bring autonomous vehicles to reality [18,21]. Through simulations, Park et al. [11] calculated curve speeds and applied them in a path-tracking algorithm.

Even though curve speed estimation has been considered in several works, only a few of them have been actually implemented and tested either in simulations or real environments [9,10,11]. Glaser et al. [9], as well as Park et al. [11] have computed the ideal speed for curves based on the analysis of geometric information, but no speed limits have been contemplated. On the other hand, Lee et al. [10] considered speed limits and curve speeds, but their implementation is made for an ACC, which is a semi-autonomous solution where the driver is able to take control of the vehicle and override appropriate speeds. Our work is targeted to be implemented as an autonomous solution for path-tracking algorithms.

## 3. Steering Control Algorithms for Path Tracking

Path tracking has been one of the main challenges for autonomous navigation for the last 30 years. Its functionality resides in computing the appropriate commands for the vehicle to track a reference trajectory as accurately as possible. One of these commands is the desired vehicle orientation (θ), which together with speed (*v*), will seek to achieve good performance.

This section provides a review of some steering control algorithms used in already tested autonomous vehicles. These methods will be evaluated in Section 5 through simulations in order to prove that their performance can be improved significantly with our proposed dynamic speed adaptation method.

### 3.1. Pure Pursuit

Geometric-based pure pursuit is one of the most basic and simple methods to compute steering wheel angle (δ) for a lateral controller. Its calculation relies on defining a goal point in a reference path according to a certain distance called the look-ahead distance ($l_{d}$), to try to reach it at every time step *t* through a circular arc.

The pure pursuit control law is given as [35]:

(1) $$\delta \left(t\right)=\arctan\left(\frac{2L\sin(\alpha (t))}{l_{d}}\right)$$

where *L* is the distance between the front axle and rear axle (wheelbase) and α is the angle between the vehicle heading vector and the look-ahead vector.

This algorithm suffers mainly from cutting corners (neglects curvature information) if look-ahead distance is big and severe oscillations if it is defined as too small.

### 3.2. Stanley Method

The Stanley steering controller was developed by the Stanford Racing Team and implemented in their autonomous vehicle (Stanley) for the DARPA Grand Challenge. Their controller is based on a non-linear feedback function based on lateral errors [12] measured from the front wheel axle.

Four parameters are considered for the steering angle (θ) computation at each time step (*t*): lateral error (ε), vehicle speed (*v*), a gain value (*k*) and the angle difference (ψ) between the vehicle and the nearest path segment orientation. Its equation is given by:

(2) $$\theta \left(t\right)=\psi \left(t\right)+\arctan\frac{k\varepsilon (t)}{v(t)}$$

This method is a simple basic approach that proved to be efficient in the DARPA race with an average lateral offset of ±74 cm.

### 3.3. Alice Method

The Caltech Team developed a control strategy for trajectory tracking based on a nonlinear control law [36]. The method was implemented in the Alice autonomous car, which took part in the DARPA Urban Challenge.

The vehicle was assumed to have Ackermann steering dynamics leading to the use of an approximation of the bicycle model. The steering wheel angle (Φ) is computed with the following formula at each time step *t*:

(3) $$\tan\left(\Phi \left(t\right)\right)=\frac{-\cos\left(e_{\theta }\left(t\right)\right)e_{⊥}\left(t\right)-\left(l_{1}+l_{2}\right)\sin\left(e_{\theta }\left(t\right)\right)}{l_{1}-\left(l_{1}+l_{2}\right)\cos\left(e_{\theta }\left(t\right)\right)+\sin\left(e_{\theta }\left(t\right)\right)e_{⊥}\left(t\right)}$$

where $e_{⊥}$ is the lateral rear axle error (cross-track error), $e_{\theta }$ is the angle between the vehicle direction and the tangent of the projected vehicle position in the path (yaw error), $l_{1}$ is the vehicle wheelbase and $l_{2}$ is the distance to the target point.

The aim of this method is to reduce $e_{⊥}$ and $e_{\theta }$, which is equivalent to measuring the lateral error from the vehicle center line. For our purposes, the results presented in Section 5 will show only the lateral error from the center of the car. This to provide a common comparison indicator when evaluating all of the considered steering wheel methods.

### 3.4. Lombard Method

Lombard et al. [37] proposed a steering controller based on the well-known pure pursuit algorithm. Their method was implemented in a Renault Scenic car and shown at the Intelligent Transportation System World Congress (2015).

The computation of the steering angle introduces a coefficient *k*, defined as 1−αS (see Equation (4)), with the aim to reduce the cutting corners effect without changing the target point distance or vehicle’s speed. In other words, this coefficient tries to attenuate the area surface (*S*) between the reference path and the arc drawn from pure pursuit. The steering angle parameter (δ) is defined as follows:

(4) $$\delta =\arctan\left(\left(1-\alpha S\right)\frac{E}{R}\right)$$

where *E* is the distance between the axles of the car, *R* is radius of the circle drawn to follow the target point and α is a coefficient set to 0.02.

Lombard’s method provides lateral errors smaller than 1 m when the speed is set to 36 km/h.

## 4. Dynamic Speed Adaptation

It is well known in the path-tracking literature that vehicle speed may increase or decrease lateral errors. In the ideal case, a good definition should lead the vehicle to track the trajectory accurately (with no errors) [38]. For this reason, most of the works have focused on defining a maximum speed (which is usually a small value [11,39]) to tune the best performance with their algorithms. Nevertheless, speed variations in different situations need to be considered to simulate human driving behavior. The objective of our proposed DSA method is to simulate this behavior.

**Figure 2:**
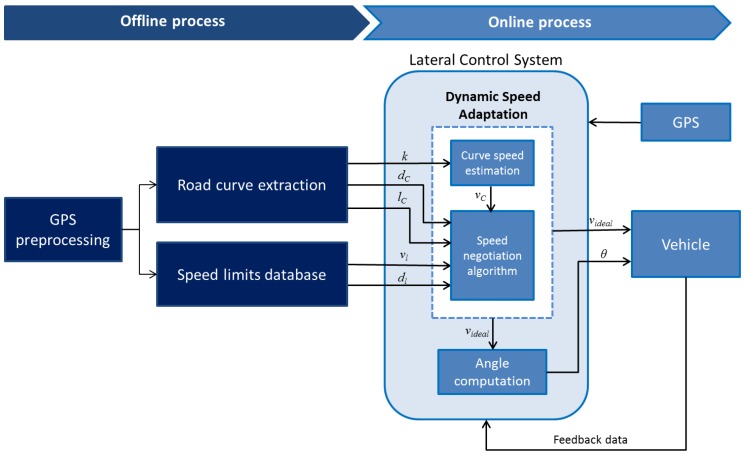
Overall data-flow of the dynamic speed adaptation module.

Our DSA approach defines the speed to be applied (through a speed negotiation algorithm) by the lateral control system in order to travel a safe trajectory. First, GPS information is preprocessed in order to remove noise and reduce the number of points. Second, road speed limits together with their corresponding positions are detected in the traveled GPS path and saved in a database. Third, sharp curves are identified along with their positions, radius and length in order to compute their convenient speeds and send them to the speed negotiation algorithm (see Figure 2). A more detailed description is presented in the following sub-sections.

### 4.1. GPS Preprocessing

Real-Time Kinematic (RTK) refers to a technique able to correct positioning information (GPS) with centimeter-level accuracy. This correction is carried out by a base station that reduces or removes positioning errors [40] caused by environmental surroundings or weather conditions.

RTK-GPS was used during data collections in order to have reliable GPS paths. Nevertheless, due to different circumstances like the loss of connection between the base and receiver, noise appeared in the GPS data. This noise was removed manually in our datasets in order to have reliable learning routes. We identified the noisy points and interpolated their new positions based on the previous and following points.

Once GPS noisy points are removed, we computed the Euclidean distance between each pair of points to keep GPS positions at an approximately 3.5-m distance between each other; this with the aim to reduce the amount of data for further processing, i.e., road curvature estimation and speed limits extraction.

### 4.2. Speed Limit Database

Speed limits vary between countries, types of roads, areas, vehicles and inclusive timing [23]. They may also be affected by certain circumstances like construction or special events around a zone (i.e., fairs, carnivals, street markets, etc.). Even though it is important to consider all possible scenarios for the speed limit database, it is very hard to maintain updated zones where temporary situations occur.

Digital maps contain speed limit information, but most of them are not free. OpenStreetMaps [41], on the other hand, provide open data with fixed speed limits (without considering temporary data), but for our traveled paths, the information is not included.

We created our own speed limits database, based on the typical manual driving situations and considering the code regulations established by the French government [24]. In France, urban areas have speed limits of 50 km/h varying to 30 or even 20 km/h in agglomeration areas, residential areas or school zones [24].

According to the recorded RTK-GPS information, we:

1. Define start and end points.
2. Identify positions where speed limits change.
3. Compute the distance ($d_{l}$) from the speed limit change point to the reference starting point in the path.
4. Save the distance ($d_{l}$) for every speed limit change position identified together with its respective speed limit value ($v_{l}$).

**Figure 3:**
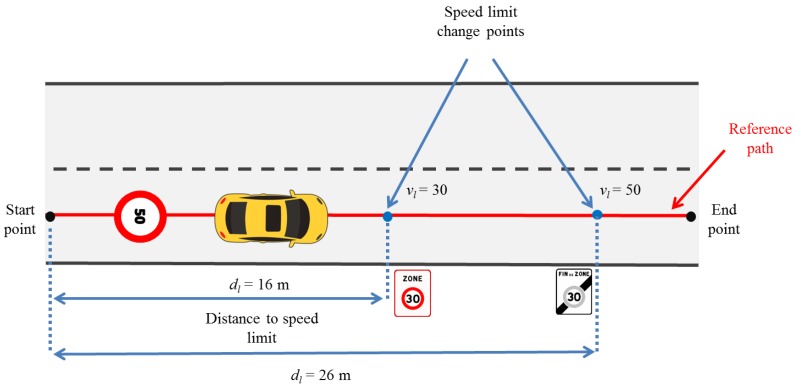
Speed limit driving scenario.

We identified positions where speed limits change from 50 km/h to 30 km/h and vice versa. By default, the speed limit at the starting point is set to 50 km/h if no other speed limit is identified. An illustration of the procedure is presented in Figure 3.

The creation of the speed limits database is performed off-line and only after GPS information is preprocessed. The description of the datasets and their speed limits identified is provided in Section 5.

### 4.3. Curve Speed Estimation

Driving through an unknown sharp curve is always risky if the speed is not adapted to control the vehicle. In real life, the driver should be able to maneuver the steering wheel and reduce speed simultaneously in order to stay in the lane and pass the curve with comfort and safety. The same principle should be applied for autonomous control systems (reduce speed before going through a curve).

In order to control the vehicle’s speed appropriately, curve identification needs to be performed for the traveled GPS path to proceed with the computation of convenient speeds for each curve. Li et al. [6] extracted and classified curves considering road data from Geographic Information Systems (GIS). GIS store different types of information providing very accurate road geometry, but this is not always available. In our work, curvature analysis is performed using GPS data following the technique in [6] with minor modifications and extending its curve estimation to identify sharp curves.

Curve analysis is a very important step for intelligent vehicle systems [42] since it will keep lateral errors as small as possible, and at the same time, it will reduce the number of crash accidents.

#### 4.3.1. Sharp Curve Estimation

Sharp curves are defined as dangerous curves if their radii are small or central angles are big [6]. This type of curve deserves special attention due to the fact that they correspond to locations where the vehicle needs to decrease speed in order to take the curve as if a human were driving, keeping small lateral errors [31].

**Figure 4:**
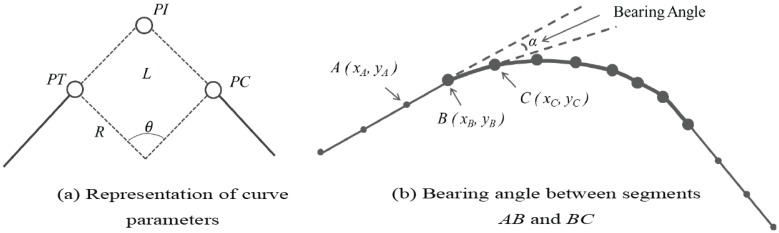
Curve parameters representation.

In order to identify sharp curves, we first localized all possible curves in the traveled GPS path based on Li et al.’s [6] work. Localizing curves means identifying their start and end points, which are also called points of tangency (PT) and points of curvature (PC), respectively. Once these points are identified, their curve characteristics can be computed: point of intersection (PI), curve length (*L*), radius (*R*) and central angle (θ). An illustration of all these characteristics is shown in Figure 4a.

Classifying points as being part of a curve or of a straight segment is the first step to be able to identify start and end points (PT and PC) in a curve. This procedure is performed by computing the bearing angle (α) between two consecutive segments formed with three points (A,B,C), as seen in Figure 4b, using Equation (5).

(5) $$\alpha =cos^{-1}\left(\frac{\left(x_{B}-x_{A}\right)\left(x_{C}-x_{B}\right)+\left(y_{B}-y_{A}\right)\left(y_{C}-y_{B}\right)}{\sqrt{{\left(x_{B}-x_{A}\right)}^{2}+{\left(y_{B}-y_{A}\right)}^{2}}\times \sqrt{{\left(x_{C}-x_{B}\right)}^{2}+{\left(y_{C}-y_{B}\right)}^{2}}}\right)\times \frac{180}{\pi }$$

Once the bearing angle is computed, a threshold value is assigned to determine if the point *B* is considered as part of a curve or not. If the angle α is bigger than the defined threshold, the center point *B* is described as being part of a curve. Choosing the right threshold is a very important step, since curvature identification depends on this value. In this work, a threshold value of $1.25^{\circ }$ was chosen as proposed in [6] after evaluating curvature identification with different values. It is worth mentioning that values smaller than 1.25 gave us false positives, as almost every point was considered as being part of a curve, and values bigger than 1.25 discarded long and smooth curves.

**Figure 5:**
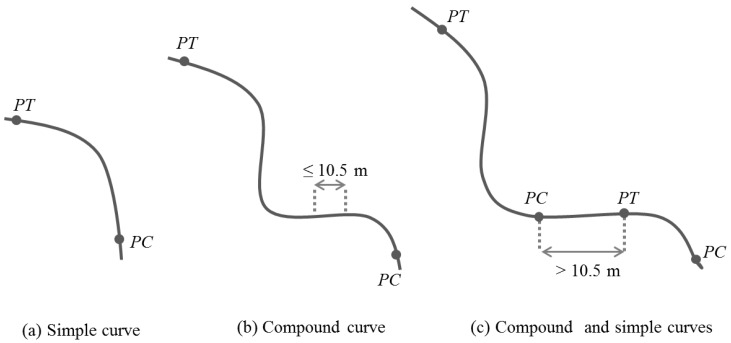
Curve classification.

Based on Li et al.’s work [6], curves were classified as simple or compound. A compound curve is a curve formed with at least two consecutive curves separated by a certain distance between each other. In our datasets, this distance was set to 10.5 m, which is the approximation of considering three consecutive points in our preprocessed GPS path. An example of the two types of curves is illustrated in Figure 5.

After each curve is defined by its start and end points (PT and PC), its geometric information, consisting of its radius (*R*), length (*L*) and central angle (θ), is computed through Equations (6) to (8). In order to perform these computations, the center point of the curve (*O*) is identified following the work [6].

(6) $$R=\sqrt{{\left(x_{PC}-x_{O}\right)}^{2}+{\left(y_{PC}-y_{O}\right)}^{2}}$$

(7) $$C=\sqrt{{\left(x_{PT}-x_{PC}\right)}^{2}+{\left(y_{PT}-y_{PC}\right)}^{2}}$$

(8) $$\theta =2\times sin^{-1}\left(\frac{C}{2R}\right)\times \frac{180}{\pi }$$

Curve length (*L*) was estimated by summing up the Euclidean distances between the segments that form the curve.

**Figure 6:**
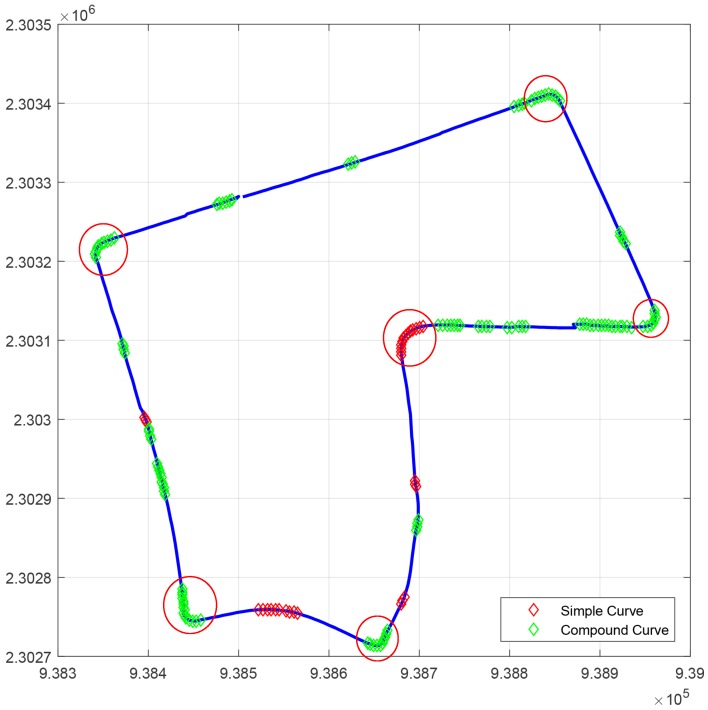
Curve classification of UTBM-2 dataset (see Section 5 for reference). Sharp curves are marked with red circles.

As we are interested in considering only dangerous curves, sharp curves were categorized if their angle ranges from $30^{\circ }$ to $180^{\circ }$, or their radius is between 5 and 18 m according to the American Association of State Highway and Transportation Officials’ (AASHTO’s) “A Policy on Geometric Design of Highways and Streets” [43]. Figure 6 shows an example of the curve classification according to [6], and marked in red circles are the sharp curves identified with our method. In this work, only sharp curves are considered to adjust the speed in path-tracking simulations.

#### 4.3.2. Speed Computation for Sharp Curves.

**Figure 7:**
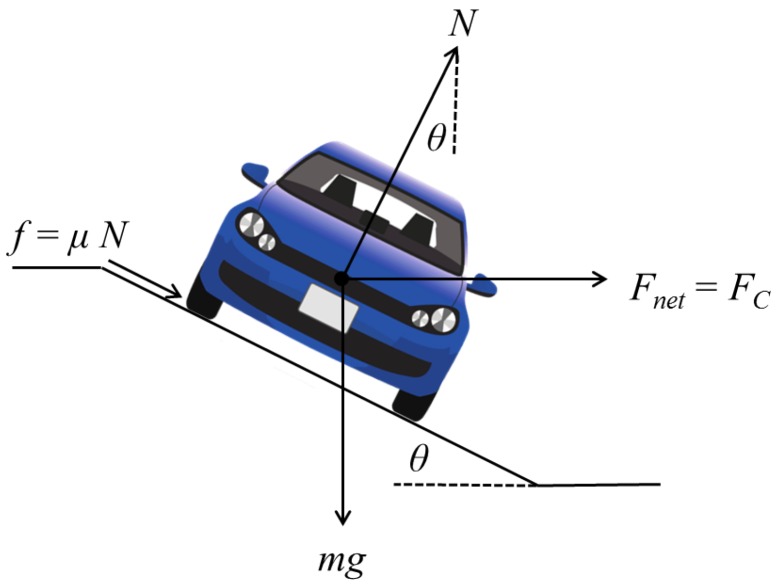
Centripetal force diagram.

Curve speed estimation depends on centripetal and centrifugal forces. Centripetal force, which relies on the friction between tires and the roadway, is the one that makes the vehicle follow the curve, while centrifugal force tries to move the vehicle outwards from the curve. In order to compensate this last force, road curves are designed with an inclination known as a banked angle (θ) or super-elevation angle (*e*). In other words, ideal speed in curves will be computed depending on the curvature of the path (*k*), friction coefficient (μ) and super-elevation angle (*e*), as shown in Figure 7, where *N* is the normal force, *f* is the friction force, μ is the friction coefficient, *m* is the vehicle mass, *g* is the gravity and $F_{net}$ is the net force.

Through the normal force (*N*), friction force (*f*) and weight force (mg) vectors, the centripetal force is defined as:

(9) $$F_{C}=\frac{mv^{2}}{r}$$

where *m* represents vehicle mass, *v* vehicle speed and *r* the curve radius.

Summing up the vertical components, normal force can be described following Equation (10).

(10) Ncosθ=mg+fsinθ

(11) $$N=\frac{mg}{\cos\theta -\mu \sin\theta }$$

The net force is calculated through the horizontal components:

(12) $$F_{net}=N\sin\theta +N\mu \cos\theta$$

Substituting the normal force in Equation (12),

(13) $$F_{net}=\left(\frac{mg}{\cos\theta -\mu \sin\theta }\right)\left(\sin\theta +\mu \cos\theta \right)=\frac{\tan\theta +\mu }{1-\mu \tan\theta }mg$$

Since the friction coefficient (μ) usually varies from 0.1 to 0.16 [11], the value in the denominator tends to be around one, so it can be discarded. Now, the net force can be defined as the centripetal force ($F_{net}=F_{centripetal}$), obtaining the following equation:

(14) $$\left(\tan\theta +\mu \right)g=\frac{v^{2}}{r}$$

Using super-elevation e=tanθ and curvature information $r=\frac{1}{k}$, we substitute both terms into Equation (14). The ideal speed will be given by Equation (15), which is exactly the same definition as in [11].

(15) $$v=\sqrt{\frac{(e+\mu )g}{k}}$$

Following this equation, convenient speeds for each sharp curve ($v_{c}$) were estimated considering a super-elevation value between 6% and 12%, which, according to the American Association of State Highway and Transportation Officials (AASHTO) [43], is the value defined for rural and urban roads. *k* is the curvature information computed as the reciprocal of the radius, $k=\frac{1}{r}$.

**Figure 8:**
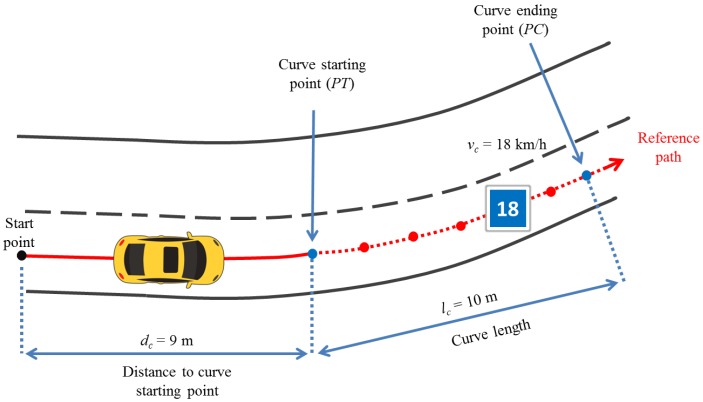
Curve driving scenario.

Finally, given a sharp curve detected in the traveled GPS path, we computed the distance ($d_{c}$) from the reference start point of the path to each curve starting point (PT) as shown in Figure 8. This distance together with its respective curve lengths ($l_{c}$) and the convenient speed ($v_{c}$) are the parameters passed to the speed negotiation algorithm, detailed in the next subsection.

### 4.4. Speed Negotiation Algorithm

In typical manual driving situations, drivers notice the upcoming speed limit sign or curve and slow down or accelerate (depending on the current driving speed) to handle the road properly. Automating this procedure in ground vehicles requires knowledge ahead to adjust the vehicle’s speed before entering a sharp curve or arriving at a new speed limit zone. Our work covered this knowledge extraction in Section 4.2 and Section 4.3.

The speed negotiation algorithm tries to simulate human driving behavior based on the knowledge extracted from curves and speed limits to decide the ideal speed ($v_{ideal}$) at which the vehicle should be traveling. This negotiation depends mainly on the actual vehicle position in the GPS path, and the analysis of the input parameters coming from curve and speed limits as shown in Figure 2.

**Figure 9:**
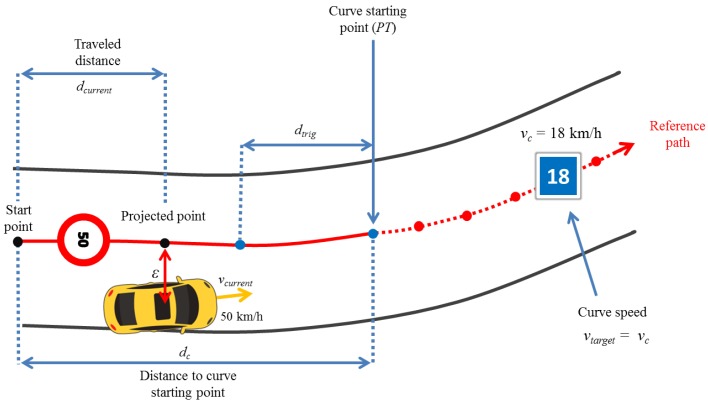
Curve driving scenario projecting vehicle position in the reference path.

Initially, the lateral control system computes the lateral error (ε) and the current vehicle traveled distance ($d_{current}$) in the path (see Figure 9). This computation is estimated by projecting the current vehicle position on the reference path through spline interpolation to match the GPS points.

Once the projected vehicle position is known, the speed negotiation algorithm analyses which type of road segment (curve or speed limits) is closer to the current vehicle position and computes a trigger distance. This trigger distance ($d_{trig}$) has the objective to take into account a smooth deceleration behavior to reach the ideal speed with comfort. It is calculated with the following equation:

(16) $$d_{trig}=\frac{v_{target}^{2}-v_{current}^{2}}{2a}$$

where:

- $v_{target}$ is either the speed limit ($v_{l}$) or curve speed ($v_{c}$),
- $v_{current}$ is the current vehicle’s speed and
- *a* is the deceleration value to be applied.

An appropriate deceleration value is considered according to a study made by Maurya et al. [44], which compares several deceleration behaviors at different speeds in various vehicle types. In our case, we set a maximum acceleration ($a_{max}$) and deceleration ($a_{neg}$) of 2 m/$s^{2}$ to provide a comfortable transition between speeds and avoid longitudinal jerks.

If sharp curved positions overlap with zones where a speed limit is lower than 50 km/h, our algorithm gives priority to the lower speed, which is usually the curve speed.

Considering all aspects discussed above, the speed negotiation algorithm works as follows:

At each time step, an ideal speed ($v_{ideal}$) results from the speed negotiation algorithm. This speed is used by the lateral control system to calculate an appropriate angle (θ) to be applied by the vehicle steering wheel. During every control cycle, the lateral control system sends to the vehicle speed and angle parameters to provide a smooth tracking performance.

The goal of reducing the speed before arriving at a curve or a speed limit zone will provide the passenger comfort, avoiding abrupt rapid decelerations. At the same time, respecting the ideal speed definition will increase safety and provide more time in case hazardous situations occur. As a result, lateral errors will decrease, which is the aim of path-tracking algorithms (shown in Algorithm 1).

**Algorithm 1: **Speed negotiation pseudo-algorithm.
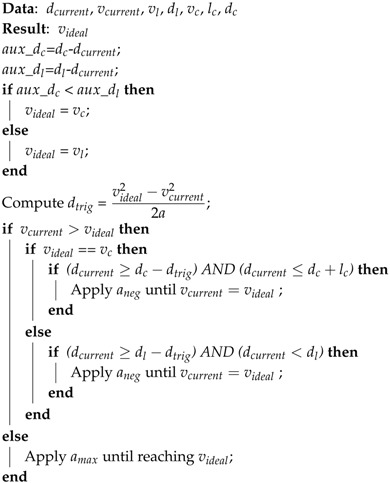


## 5. Results

In this section, we perform simulations with different lateral control algorithms on a simulated path and real datasets. This is with the aim to show how tracking accuracy is improved if correct speed definitions are taken into account for each segment in the traveled path. Consequently, a sense of comfort and safety will be provided to the passenger, reducing traffic accidents and severe injuries [1].

**Figure 10:**
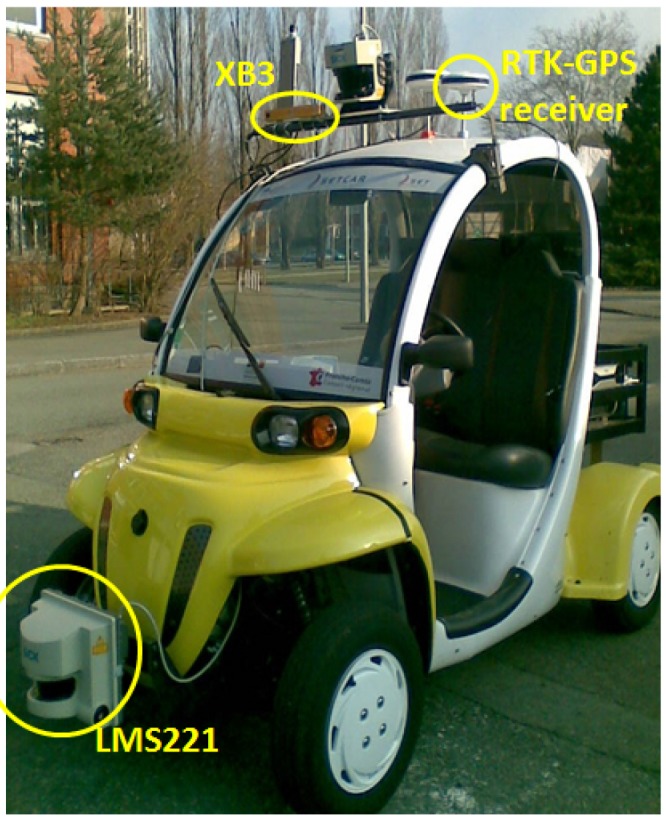
Equipped experimental platform.

Three paths (one created and two real) were considered for the simulations. Each path has different length, curve characteristics and speed limits. The real datasets (traveled paths) were acquired by Qiao et al. [45] with the experimental platform shown in Figure 10 in Belfort, France. The data of the paths are based only on GPS information captured by GPS and RTK-GPS receivers. The simulated path was created with the aim to compare how noise affects the tracking performance of lateral control algorithms.

**Figure 11:**
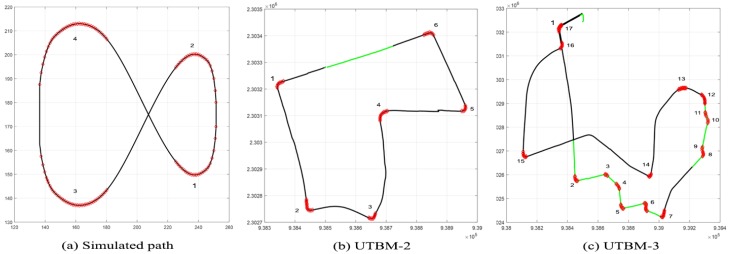
Datasets with sharp curves and speed limits marked. The normal black line represents a speed limit of 50 km/h, the green line a speed limit of 30 km/h and the sharp curves in red.

A visual representation of the considered paths can be seen in Figure 11. They are briefly presented below.

1. Simulated path: This path is composed of 210 continuous points in a figure eight shape form. Its total distance is about 374 m with four sharp curves identified. Since it is a virtual path, we set the speed limit for the entire track to 50 km/h, as this is the default speed limit in urban areas in France [24].
2. UTBM-2 dataset: The traveled path contains 541 points after the preprocessing step performed as described in Section 4.1. The distance of this track is about 2.2 km with detected speed limits of 30 km/h in school zones. Six sharp curves were identified with different geometric characteristics.
3. UTBM-3 dataset: This path is the longest one with a distance of about 4.5 km. It is represented by 1136 points after the preprocessing step. The number of sharp curves (dangerous curves) detected was 17. The speed limits for this path are 30 km/h in residential areas and 50 km/h otherwise.

Different path tracking simulations were performed through the tool developed by Lombard et al. [37]. These simulations include the analysis of different lateral control algorithms with and without considering the ideal speeds obtained by our DSA method.

In order to compare the performance of our proposed DSA method between the different steering control algorithms, (1) Pure Pursuit (PP), (2) Stanley, (3) Alice and (4) Lombard, we compute the root mean square error ($e_{rms}$) per each dataset for each of the four algorithms as follows:

(17) $$e_{rms}=\sqrt{\frac{\sum {\left(p_{d}\left(t\right)-p_{c}\left(t\right)\right)}^{2}}{m}}$$

where $p_{d}$ and $p_{c}$ represent the desired and current vehicle position, respectively. The difference between $p_{d}$ and $p_{c}$ is the lateral error, while *m* is the total number of sampling results every 400 ms (latency of the system). Then, the average error on the total number of datasets per each method is calculated as:

(18) $$\bar{e_{rms}}=\frac{1}{N}\sum e_{rms}$$

where *N* is the number of datasets per algorithm to be evaluated. In the case of curve evaluation, *N* equals three (two real and one simulated datasets), while for speed limits, *N* equals two (the two real datasets).

In France, the speed limit in urban areas is defined as 50 km/h [24], which is the maximum speed assigned in the simulations. Other than that, in our datasets, speed limit zones of 30 km/h were identified and considered to adjust vehicle’ speed. Regarding curve speeds, they vary from 14 to 36 km/h depending on the sharpness detected for each curve.

**Figure 12:**
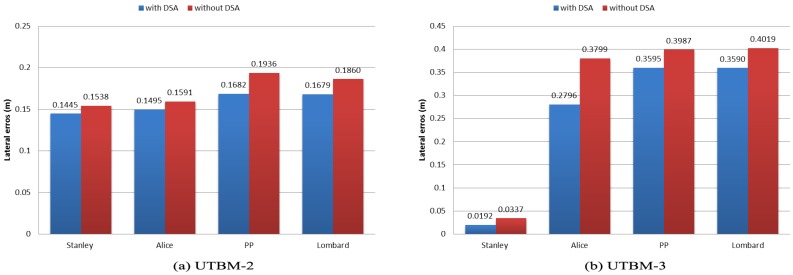
Root mean square error ($e_{rms}$) comparison of lateral errors obtained in 30 km/h speed limit segments.

We analyzed lateral errors produced when speed limits change from 50 to 30 km/h in the UTBM-2 and UTBM-3 datasets (see Table 1). In other words, we analyzed lateral errors in segments of 30 km/h (green zones as seen in Figure 11) and compare the results for each lateral control method. The speed limit zone of 30 km/h in UTBM-2 is a straight segment, and even if lateral errors in all methods are similar (15 to 20 cm error), the performance of all methods is improved considering our DSA method (see Figure 12a). Lateral errors in the UTBM-3 dataset vary more due to its road geometry; therefore, improvements with our DSA method are more noticeable, as shown in Figure 12b.

**Figure 13:**
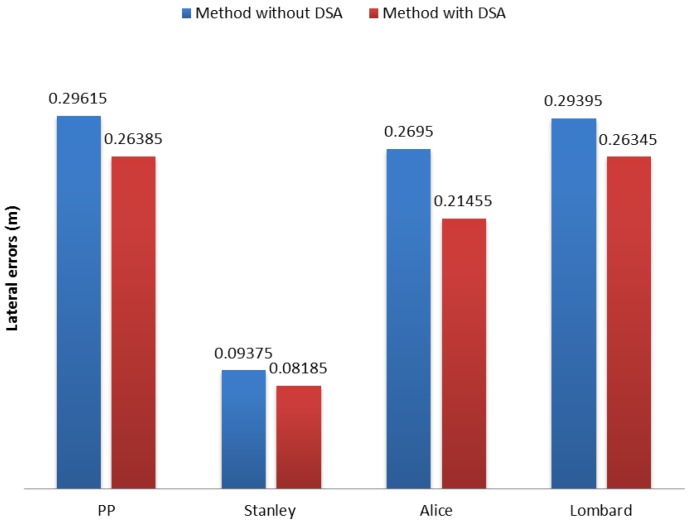
Average root mean square error ($\bar{e_{rms}}$) of lateral error comparison between methods for 30 km/h speed limit segments.

On average, the evaluation of speed limits in segments of 30 km/h improved all of the methods with our DSA, but not more than 5 cm, as seen in Figure 13. It is worth mentioning that the literature has proven that respecting speed limits increases safety [2,3,4,19]. Autonomous vehicles capable of adjusting automatically speed limits will be preferred, since, in real scenarios, drivers go beyond the authorized speeds, being prone to cause accidents.

Now, we will focus our analysis on the most dangerous segments, which are the sharp curves. Table 2, Table 3 and Table 4 show lateral error results obtained by the different steering control algorithms in the detected curves for each path.

**Figure 14:**
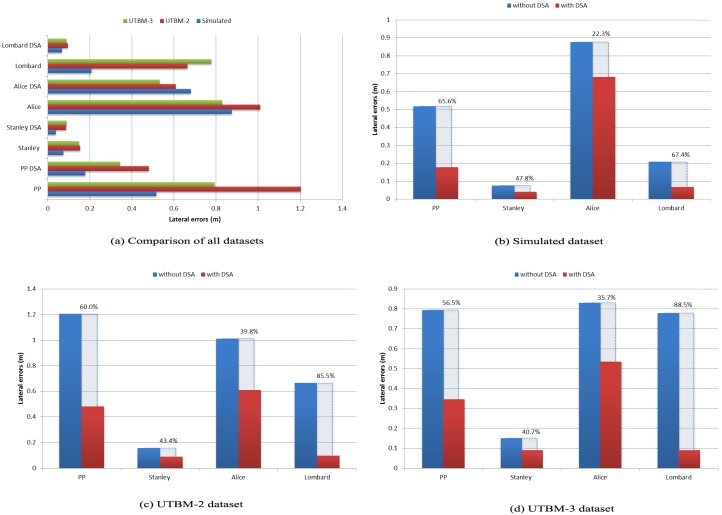
Root mean square error ($e_{rms}$) comparison of lateral errors obtained in sharp curves for each dataset.

We will start comparing the simulations performed between paths that contain or not noisy information. Regarding tracking error results obtained in the simulated path (Table 2), we can see that most of the lateral errors are the smallest (except for the Alice method) compared to errors obtained in real datasets (Table 3 and Table 4). A visual representation of this comparison can be seen in Figure 14a. We can confirm that noise is an important factor for tracking algorithms, and it deserves special attention to reduce it before controlling autonomous cars. In the literature, the authors have combined different information to deal with noise, e.g., taking into account the dynamics of the car (position, orientation, speed) [20].

**Figure 15:**
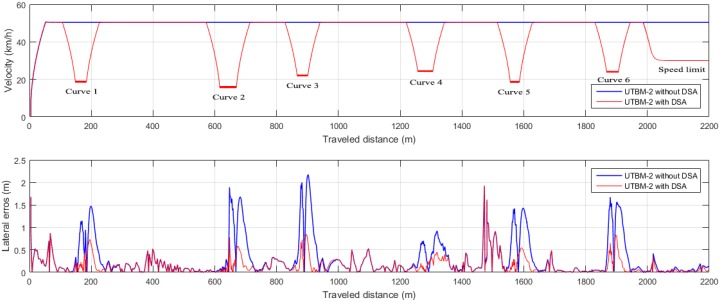
Speed (**top graph**) and lateral error (**bottom graph**) comparison in the UTBM-2 dataset using the PP method with and without DSA.

The improvements of each method, with and without considering speed regulations in curves, range from 22% to 88% depending on the path. For example, in the simulated path (see Figure 14b), tracking error is reduced by 19 cm in the Alice method (22% improvement). In UTBM-2, the method with the largest root mean square error is pure pursuit (1.2 m), reduced by 72 cm with our DSA (see Figure 14c). A visual representation of lateral errors on the UTBM-2 dataset with Pure Pursuit method is shown in Figure 15, which illustrates how our DSA method is able to reduce errors significantly in sharp curves. At the same time, it is clearly noticeable that the biggest lateral errors are present in sharp curves confirming the need to adapt vehicle’ speed. Furthermore, in the UTBM-3 dataset (see Figure 14d), tracking error is reduced by about 45 cm in the pure pursuit method, while for the Lombard method, it is 69 cm (88% improvement).

**Figure 16:**
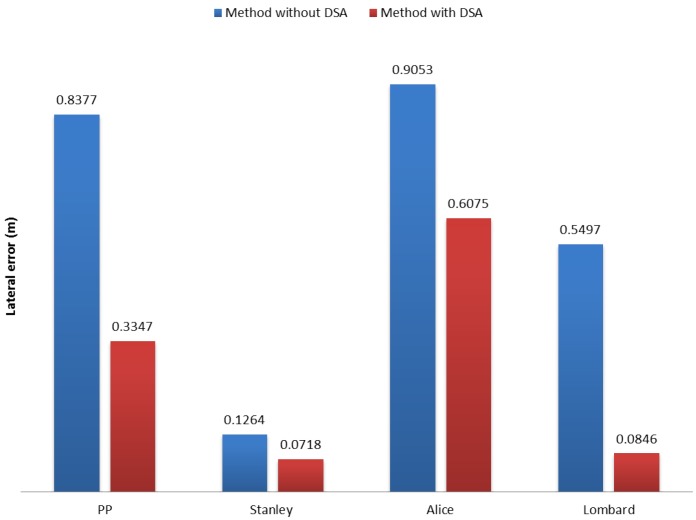
Average root mean square error ($\bar{e_{rms}}$) of the lateral error comparison between methods for sharp curves.

In general, the performance of our DSA method in sharp curves showed significant (at least 10-cm error) improvements for most of the methods (see Figure 16), except for the Stanley method (improvement of around 5 cm). The reason behind this behavior is the definition of its goal point. Since the Stanley method is based on Pure Pursuit (PP) and the goal point is defined with a short distance, it does not suffer greatly from the “cutting corners” effect.

As tracking errors for speed limits decrease, but not significantly (1 cm for Stanley, 5 cm for Alice and 3 cm for pure pursuit and Lombard), we will base our final conclusion on the results obtained in sharp curves. Tracking errors with our DSA method improved three methods significantly, Pure Pursuit (PP), Alice and Lombard. Even though the improvements for the Stanley method are not considered significant, there is an improvement of about 5 cm when speed adaptation is used. The two methods that benefit the most with DSA are pure pursuit and Lombard (as seen in Figure 16) with about a 50- and a 47-cm difference respectively for sharp curves. For the Alice method, the difference is about 30 cm, which is also a very good improvement. These results prove that, having the adapted speeds to pass through sharp curves certainly leads to provide the passenger confidence and safety for taking a ride in an autonomous vehicle.

One of the main limitations for our proposed method resides in GPS noise. Nevertheless, this limitation can be treated combining more information, for example vehicle dynamics [20], to analyze position, orientation and speed. Inaccurate curvature geometry extraction is another limitation when noisy GPS information is present. Nonetheless, this last problem can be overcome extracting curve parameters from digital maps.

## 6. Conclusions

We have proposed a real-time Dynamic Speed Adaptation (DSA) method based on the analysis of speed limits and curvature information. A speed limit database of the traveled paths was created and considered as input for our DSA. Curvature information extraction allowed DSA to identify sharp curves and to compute the recommended speed for each one. The speed limit database and curve geometric information are extracted off-line from GPS paths in order to provide knowledge ahead of the road while traveling in it.

The tracking performance using our approach showed significant reduction of lateral errors. This improvement may potentially prevent accidents and reduce severe injuries in real driving scenarios, which is exactly the aim of any autonomous vehicle.

The main advantage of our proposed DSA method is its adaptability, since it can be implemented in the vehicle lateral control system independently of the steering wheel angle computation. Besides, it enables the car to drive with recommended speeds through straight and curved segments respecting traffic regulations. Furthermore, it is smart enough to localize upcoming sharp curves and apply a comfortable deceleration value before arriving at them, reaching their ideal speeds to traverse them.

Future works will include the collection and analysis of vehicle dynamics in order to deal with GPS noise. This information will correct noisy points in the path by comparing the vehicle’ speed and orientation with the GPS data.

Furthermore, we will incorporate the information of a stereo system in order to bring perception capabilities to the autonomous vehicle. The information of this sensor together with a semantic segmentation approach will allow us to: (1) detect the road if GPS is not available; (2) identify speed limit traffic signs in case no speed limit database is accessible; (3) recognize lane markings on the road to guide the algorithm for road boundary detection and shape estimation; and (4) prevent the vehicle from turning if the lane where it is driving has some curved arrows painted. Detecting and recognizing lane markings on the road are a potential source of information for the autonomous car, since they provide very useful knowledge to learn driving behaviors.

## Figures and Tables

**Table 1:** Error comparison in zones of 30 km/h with different lateral control methods. Results are expressed in meters. DSA, Dynamic Speed Adaptation; PP, Pure Pursuit.

Dataset	Stanley	Stanley DSA	Alice	Alice DSA	PP	PP DSA	Lombard	Lombard DSA
UTBM-2	0.0182	0.0158	0.0452	0.0416	0.1005	0.0554	0.0845	0.0550
UTBM-3	0.0210	0.0112	0.2840	0.1934	0.3346	0.2823	0.3374	0.2817
Average	0.0196	0.0135	0.1646	0.1175	0.2175	0.1688	0.2109	0.1683

**Table 2:** Error comparison in sharp curves of different Lateral Control methods for the simulated path. Results are expressed in meters.

Curve	Stanley	Stanley DSA	Alice	Alice DSA	PP	PP DSA	Lombard	Lombard DSA
1	0.0599	0.0388	0.7637	0.6249	0.1935	0.2257	0.1854	0.0613
2	0.0762	0.0302	0.8286	0.6128	0.7972	0.0603	0.1752	0.04807
3	0.1029	0.0508	1.2195	0.9323	0.4351	0.2239	0.2223	0.09659
4	0.0493	0.0315	0.5757	0.4726	0.2107	0.1204	0.1531	0.04335
Average	0.0721	0.0378	0.8469	0.6607	0.4091	0.1576	0.1840	0.06233

**Table 3:** Error comparison in sharp curves of different lateral control methods for the UTBM-2 dataset. Results are expressed in meters.

Curve	Stanley	Stanley DSA	Alice	Alice DSA	PP	PP DSA	Lombard	Lombard DSA
1	0.0722	0.0288	0.5747	0.3308	0.8657	0.2451	0.4231	0.0552
2	0.0743	0.0245	0.6572	0.4945	0.7135	0.2372	0.5050	0.0589
3	0.2586	0.1797	1.2132	0.7815	1.5847	0.6940	0.9028	0.1292
4	0.0574	0.0265	0.5724	0.2591	0.5528	0.2396	0.2616	0.0382
5	0.1214	0.0449	1.0485	0.5650	1.0909	0.3627	0.5347	0.0751
6	0.1001	0.0441	0.9109	0.4528	1.4346	0.7075	0.7563	0.0687
Average	0.114	0.0581	0.8295	0.4807	1.0404	0.4143	0.5639	0.0709

**Table 4:** Error comparison in sharp curves of different lateral control methods for the UTBM-3 dataset. Results are expressed in meters.

Curve	Stanley	Stanley DSA	Alice	Alice DSA	PP	PP DSA	Lombard	Lombard DSA
1	0.0763	0.0328	0.6984	0.3589	0.5012	0.2007	0.4840	0.0574
2	0.0910	0.0311	0.7711	0.4342	0.5934	0.1141	0.5959	0.0530
3	0.0545	0.0258	0.3409	0.2619	0.4462	0.1409	0.4498	0.0337
4	0.0421	0.0196	0.3409	0.1912	0.4056	0.0975	0.3857	0.0218
5	0.0893	0.0296	0.7401	0.4422	0.6162	0.0948	0.6225	0.0519
6	0.4570	0.4209	1.4465	1.0717	1.3936	1.3865	1.4099	0.2663
7	0.1697	0.1054	1.0596	0.6294	0.8554	0.565	0.8769	0.0835
8	0.1781	0.0705	1.1031	0.7269	0.9603	0.3007	0.9640	0.0818
9	0.0718	0.0297	0.3765	0.3331	1.1918	0.3802	1.2084	0.0523
10	0.0753	0.0278	0.5634	0.3694	1.0366	0.1566	1.0426	0.0488
11	0.0344	0.0183	0.2441	0.1671	0.5754	0.4024	0.5825	0.0240
12	0.0472	0.0247	0.4376	0.2123	0.2039	0.1248	0.2053	0.0306
13	0.0209	0.0193	0.2167	0.0885	0.1104	0.1312	0.1101	0.0145
14	0.1573	0.0669	0.9394	0.6268	0.9873	0.1431	0.9973	0.0613
15	0.1027	0.0389	0.9034	0.4698	0.5558	0.2250	0.5589	0.0651
16	0.1171	0.0424	0.7964	0.4465	0.9738	0.3490	0.8710	0.0706
17	0.1505	0.0615	0.5531	0.6594	0.934	0.2574	0.8212	0.0813
Average	0.1138	0.0627	0.6783	0.4405	0.7259	0.2982	0.7168	0.0646
